# Enhancing sustainable innovation in AI companies: the role of perceived organizational support, job satisfaction, and job embeddedness

**DOI:** 10.3389/fpsyg.2025.1689933

**Published:** 2025-11-13

**Authors:** Fangzhou Wang

**Affiliations:** College of Public Administration, Huazhong Agricultural University, Wuhan, China

**Keywords:** perceived organizational support, job satisfaction, job embeddedness, innovation performance, AI companies, company size

## Abstract

**Introduction:**

With the rapid advancement of the artificial intelligence (AI) industry, the demand for employee innovation performance has become increasingly critical for enterprise competitiveness. However, how organizational factors such as perceived organizational support (POS) influence innovation performance among AI employees remains underexplored.

**Methods:**

Drawing upon Social Exchange Theory and Self-Determination Theory, this study develops and empirically tests a moderated mediation model. Data were collected through a questionnaire survey of employees from AI enterprises of varying sizes (*n* = 536). The model examines the mediating effects of job satisfaction and job embeddedness, as well as the moderating effect of company size, on the relationship between POS and innovation performance.

**Results:**

The findings reveal that perceived organizational support indirectly enhances innovation performance through job satisfaction and job embeddedness. The direct effect of POS on innovation performance was not significant, indicating a fully mediated relationship. Moreover, company size significantly moderated the link between POS and job satisfaction, with the effect being stronger in smaller firms.

**Discussion:**

These results highlight the importance of employee attitudes as psychological mechanisms translating organizational support into innovative outcomes. The study provides theoretical insight into how POS functions differently across firm sizes and offers practical implications for tailoring HR strategies—such as enhancing perceived support and fostering job satisfaction—to strengthen innovation capacity in AI enterprises.

## 1 Introduction

With the rapid advancement of artificial intelligence (AI), innovation has become a decisive factor in sustaining competitiveness for technology-driven enterprises. In AI companies, employees’ creative and innovative capacities determine not only organizational success but also industry leadership. However, despite increasing attention to innovation management, many firms still struggle to effectively stimulate and retain employees’ innovative potential.

Previous studies have identified both individual and organizational factors influencing innovation. While individual factors such as psychological capital and self-efficacy (Li and Zheng, 2014) play important roles, organizational factors—particularly perceived organizational support (POS)—are increasingly recognized as key determinants of innovation-related behaviors (Scott and Bruce, 1994; Rattanawichai et al., 2023). POS refers to employees’ perception of the extent to which their organization values their contributions and cares about their well-being. A high level of POS fosters positive work attitudes and proactive behaviors, ultimately enhancing individual and organizational performance (Muse and Stamper, 2007; Norizan et al., 2024).

Nevertheless, most research has focused on the direct relationship between POS and innovation performance, while the underlying psychological and behavioral mechanisms remain insufficiently explored. In particular, how job satisfaction and job embeddedness mediate this relationship—and whether contextual factors such as company size moderate it—requires further investigation.

To address these gaps, this study integrates Social Exchange Theory (SET) and Self-Determination Theory (SDT) to construct a comprehensive framework explaining how perceived organizational support (POS) influences employees’ innovation performance in AI enterprises. SET highlights reciprocal norms and extrinsic motivational processes, proposing that employees who perceive organizational care and recognition tend to reciprocate with greater commitment and creativity (Eisenberger et al., 1986). In contrast, SDT emphasizes intrinsic motivation derived from the satisfaction of autonomy, competence, and relatedness needs (Deci and Ryan, 2000). Integrating these two perspectives provides a more holistic understanding of how POS stimulates both external obligation and internalized self-motivation. This integration is particularly relevant in AI companies, where innovation occurs in highly dynamic, knowledge-intensive, and uncertain environments. In such contexts, employees must rely not only on reciprocal support mechanisms (as proposed by SET) but also on intrinsic motivation (as explained by SDT) to sustain continuous innovation. By combining SET and SDT, the present study deepens the theoretical understanding of the motivational mechanisms linking organizational support to innovation and offers practical insights for fostering sustainable innovation in technology-driven contexts.

## 2 Hypothesis development

### 2.1 Perceived organizational support and innovation performance

Perceived Organizational Support (POS) has long been regarded as a fundamental construct in organizational behavior research, representing employees’ belief that their organization values their contributions and cares about their well-being (Eisenberger et al., 1986). Within innovation-driven enterprises, POS plays a particularly salient role. In the context of artificial intelligence (AI) companies, POS represents not only recognition and care but also tolerance for failure, encouragement of experimentation, and assurance of resource support—key elements that foster employees’ confidence to innovate in environments characterized by high uncertainty and rapid technological change. By fostering a sense of security and reciprocity, organizational support encourages employees to engage more proactively in innovative endeavors.

Grounded in Social Exchange Theory (SET), POS operates through reciprocal norms that shape employees’ attitudinal and behavioral responses. When employees perceive genuine support—such as recognition, care, and encouragement—from their organization, they are more inclined to reciprocate by demonstrating greater commitment and engaging in behaviors that advance organizational objectives, including innovation (Volery and Tarabashkina, 2021). Elevated levels of POS thus enhance employees’ sense of obligation and identification with the organization, which, in turn, strengthens their motivation to contribute creatively.

Innovation performance, however, also depends on the availability of sufficient organizational resources and conducive conditions (Smale, 2016). Employees are more likely to transform creative ideas into concrete outcomes when they perceive that their organization provides adequate material support, autonomy and flexibility for experimentation. POS reflects these perceptions by signaling that the organization invests in its employees’ professional growth and innovation efforts.

Furthermore, POS mitigates employees’ perceived risks associated with innovation (Richardson et al., 2008) and fosters trust in both organizational systems and leadership. Such trust facilitates psychological safety, risk-taking, and persistence in creative problem-solving. Collectively, these factors enhance both individual and collective innovation outcomes.

Based on the foregoing theoretical rationale and empirical evidence, this study posits that employees who perceive higher levels of organizational support are more likely to exhibit stronger innovative behavior and achieve superior innovation performance.

*H*1. Perceived organizational support is positively associated with employee innovation performance.

### 2.2 Perceived organizational support and innovation performance

Perceived Organizational Support (POS) can influence employees’ innovation performance indirectly through attitudinal and affective mechanisms such as job satisfaction and job embeddedness. Drawing upon Self-Determination Theory (SDT), employees’ motivation can be categorized into intrinsic and extrinsic forms. Intrinsic motivation arises from genuine interest or enjoyment in the task itself, representing a form of autonomous behavior, whereas extrinsic motivation is contingent upon the perceived relationship between one’s actions and the expected external outcomes, such as rewards or recognition (Deci and Ryan, 2013; Gagné and Deci, 2005). SDT provides a robust theoretical framework to explain how external organizational factors—such as perceived support—can be internalized by employees, thereby transforming external motivation into self-determined motivation. This transformation offers a valuable lens for understanding how POS stimulates employees’ innovative behaviors and performance.

Intrinsic motivation is fueled by three fundamental psychological needs: autonomy, competence, and relatedness. When employees perceive that their organization satisfies these needs, they are more likely to develop positive emotional attachment, a stronger sense of belonging, and higher job satisfaction (Di Maggio et al., 2019; Fernandez and Moldogaziev, 2015). Specifically, organizational support fulfills employees’ psychological needs by providing emotional care, interpersonal trust, and social connectedness. Opportunities for collaboration, open communication, and participatory decision-making can further reinforce employees’ sense of relatedness and professional efficacy (Steindórsdóttir et al., 2020). Moreover, organizations that invest in employee development—through professional training, skill enhancement, and career advancement opportunities—enhance employees’ perceived competence, thereby strengthening their embeddedness within the organization (Bergiel et al., 2009). In this sense, POS does not merely improve immediate job satisfaction but also deepens employees’ affective and structural bonds with the organization.

Job satisfaction reflects employees’ overall evaluative judgment of their job experience, encompassing cognitive and emotional responses to various aspects of their work environment. Employees who experience high levels of satisfaction tend to demonstrate greater commitment, creativity, and persistence when facing challenges, which in turn enhances their innovative performance (Park and Rahmani, 2020; Saleh and Haidar, 2022). By contrast, low job satisfaction may inhibit idea generation and risk-taking, two key drivers of innovation.

Job embeddedness, on the other hand, represents the extent to which employees are enmeshed in the social and professional fabric of their organization. It encompasses fit (compatibility between individual and organizational values), links (the quantity and quality of interpersonal connections), and sacrifice (the perceived costs of leaving the organization). When POS strengthens these components—by promoting mutual trust, collegiality, and career investment—employees are less likely to disengage from the organization and more willing to contribute to collective innovation goals. A higher degree of embeddedness not only reduces turnover intentions but also sustains innovative efforts by fostering stability and long-term organizational commitment (Ng and Feldman, 2010).

Taken together, both job satisfaction and job embeddedness serve as vital psychological mechanisms linking perceived organizational support to innovation performance. Employees who perceive their organization as supportive tend to experience greater satisfaction with their roles and become more deeply embedded in their organizational environment, both of which enhance creative engagement and innovation outcomes.

Based on the above reasoning, this study proposes that POS positively predicts both job satisfaction and job embeddedness, and that these two variables, in turn, positively predict employees’ innovation performance.

*H*2. Perceived organizational support is positively associated with job satisfaction.

*H*3. Perceived organizational support is positively associated with job Embeddedness.

*H*4. Job satisfaction is positively associated with innovation performance.

*H*5. Job embeddedness is positively associated with innovation performance.

### 2.3 The chain mediating effect of job satisfaction and job embeddedness in organizations

Within the framework of Social Exchange Theory (SET), job satisfaction and job embeddedness can be conceptualized as key attitudinal and behavioral mechanisms through which perceived organizational support (POS) influences employees’ innovation performance. When employees perceive that their organization values their contributions and provides adequate support, they tend to experience stronger job satisfaction—a positive emotional evaluation of their work environment, responsibilities, and interpersonal relationships. In turn, this satisfaction encourages a deeper psychological and behavioral attachment to the organization, reflected in greater job embeddedness (Rayton and Yalabik, 2014).

In the context of artificial intelligence (AI) companies, these mechanisms are particularly salient. The AI industry is characterized by high work intensity, rapid technological change, and strong competition for talent, which place constant cognitive and emotional demands on employees. Under such conditions, job satisfaction reflects not only emotional well-being but also employees’ perceived recognition, autonomy, and opportunities for growth, while job embeddedness captures their long-term psychological attachment and resistance to mobility in highly fluid labor markets. Thus, both constructs play crucial roles in stabilizing innovative teams and sustaining creative engagement in AI-based organizations.

Job satisfaction represents an overall sense of fulfillment and well-being derived from one’s professional role. It encapsulates employees’ cognitive and affective evaluations of multiple job-related dimensions, including task variety, autonomy, managerial support, and working conditions. In the context of AI companies, where cognitive demands, workload intensity, and innovation pressure are exceptionally high, job satisfaction reflects not only employees’ emotional well-being but also their perceived recognition, autonomy, and opportunities for continuous learning. These elements are critical for sustaining creativity and preventing innovation fatigue in dynamic, technology-driven environments. Higher satisfaction fosters a more positive outlook toward the organization, motivating employees to reciprocate the organization’s support through enhanced commitment, loyalty, and engagement in innovation-oriented tasks.

Job embeddedness, by contrast, reflects the cumulative extent to which employees are connected to and entrenched within their organizational and social environments. It comprises three dimensions: fit, referring to the compatibility between personal values and organizational culture; links, capturing the quantity and quality of relationships within and outside the organization; and sacrifice, denoting the perceived costs associated with leaving one’s current job. In AI enterprises, characterized by rapid technological change, intense competition, and frequent job mobility, job embeddedness plays a critical role in retaining high-skilled talent and maintaining team stability. Employees who are deeply embedded in their organizations are more likely to preserve knowledge continuity, collaborate effectively, and persist in long-term innovative efforts despite environmental uncertainty. Employees with high satisfaction levels are more likely to perceive stronger person–organization fit, cultivate supportive professional networks, and recognize greater personal and career-related sacrifices associated with turnover.

According to the organizational commitment model, when employees experience satisfaction and alignment with their organizational values, they develop affective, continuance, and normative commitment (Guzeller and Celiker, 2020; Pratama et al., 2022). Affective commitment reflects emotional attachment, continuance commitment stems from recognition of the tangible and intangible benefits of staying, and normative commitment arises from moral obligation and reciprocity norms. These forms of commitment collectively reduce turnover intentions and enhance employees’ embeddedness by strengthening their psychological contract with the organization. Therefore, job satisfaction acts as a psychological foundation for job embeddedness: satisfied employees are more inclined to identify with organizational values, invest in social ties, and remain within the organization. This process illustrates a sequential path in which satisfaction precedes embeddedness.

In this sense, job satisfaction functions as an antecedent of job embeddedness, establishing a chain mediating pathway through which organizational support translates into higher innovation performance. Employees who are satisfied with their work are more likely to remain engaged, develop meaningful connections within the organization, and contribute innovative ideas that align with organizational goals. The synergy between satisfaction and embeddedness thus not only enhances retention but also promotes a sustainable climate for innovation.

Based on this theoretical reasoning and prior empirical findings, this study posits that job satisfaction and job embeddedness jointly mediate the relationship between perceived organizational support and employees’ innovation performance, forming a sequential or chain mediating effect.

*H*6. Job satisfaction and job embeddedness sequentially mediate the relationship between perceived organizational support and employees’ innovation performance.

### 2.4 The moderating effect of company size

In addition to attitudinal and affective mechanisms, contextual factors such as company size may play an important moderating role in shaping the relationships among perceived organizational support (POS), job satisfaction, and job embeddedness. From the perspective of Social Exchange Theory (SET), organizational scale influences both the nature and intensity of exchange relationships between employees and their organizations. Larger organizations, by virtue of their greater access to financial and human capital resources, are typically able to provide employees with more structured training programs, professional development opportunities, and material support (Singh, 2021). These resources can, in turn, enhance employees’ perceptions of organizational care and investment, fostering higher levels of job satisfaction and a stronger sense of embeddedness within the organization.

However, the relationship between company size and employee experience is not always linear or uniformly positive. Research has shown that the bureaucratic structures and complex hierarchies characteristic of large organizations may impede communication, constrain autonomy, and weaken interpersonal connections—factors that are closely linked to job satisfaction and embeddedness (Tansel and Gazioglu, 2022; Uhlíř and Řehoř, 2021). In such contexts, employees may experience reduced psychological safety and a diminished sense of belonging, resulting in lower satisfaction levels despite the availability of formalized support systems. Conversely, smaller organizations often offer more flexible structures, closer interpersonal relationships, and greater task variety, which can enhance employees’ emotional attachment and strengthen their job embeddedness.

Nonetheless, small organizations may also face challenges in maintaining sustained organizational support due to limited resources and less formalized development systems. As a result, employees in smaller firms may perceive lower levels of institutional support, even if relational and social bonds are stronger. These inconsistencies in the empirical literature suggest that the effects of perceived organizational support on job satisfaction and job embeddedness may differ according to company size, making it a potential boundary condition in the POS–attitude–behavior relationship.

In the context of artificial intelligence (AI) enterprises, company size is particularly influential in shaping how employees perceive and respond to organizational support. The AI industry’s rapid technological change and talent competition mean that large firms, while resource-rich, often face bureaucratic constraints that limit flexibility and psychological safety. Smaller AI companies, by contrast, tend to foster closer communication and trust but may lack formalized support systems. These contrasts make company size a key contextual factor in how perceived organizational support translates into satisfaction, embeddedness, and innovation performance.

Some scholars therefore argue that company size should not be conceptualized as a direct determinant of job satisfaction or embeddedness, but rather as a moderating variable that shapes the strength and direction of these associations (Ginting et al., 2024). Specifically, the degree to which employees perceive and internalize organizational support may depend on the organizational context in which such support is delivered. In large firms, formal systems and abundant resources may amplify the positive effects of POS on satisfaction and embeddedness. In contrast, in smaller organizations, the impact of POS may be intensified through informal, relational, and personalized exchanges that foster trust and mutual respect.

Moreover, company size also influences employees’ perceived costs of leaving the organization—a key dimension of job embeddedness. Coetzer et al. (2017) found that employees in smaller firms often experience greater psychological and social losses when considering turnover, as they tend to have closer ties to colleagues and a higher sense of individual impact. This dynamic suggests that company size not only moderates how employees perceive support but also alters how that perception translates into affective and behavioral outcomes.

Taken together, these insights highlight that company size constitutes a critical contextual factor in the exchange relationship between employees and their organizations. Recognizing its moderating influence contributes to a more nuanced understanding of how perceived organizational support operates across diverse organizational settings.

Based on the above theoretical reasoning and empirical evidence, this study aims to explore the moderating role of company size in the proposed relationships and proposes the following hypotheses:

*H*7. Company size prospectively moderates the impact of perceived organizational support on job satisfaction.

*H*8. Company size prospectively moderates the impact of perceived organizational support on the job embeddedness.

**Figure 1:**
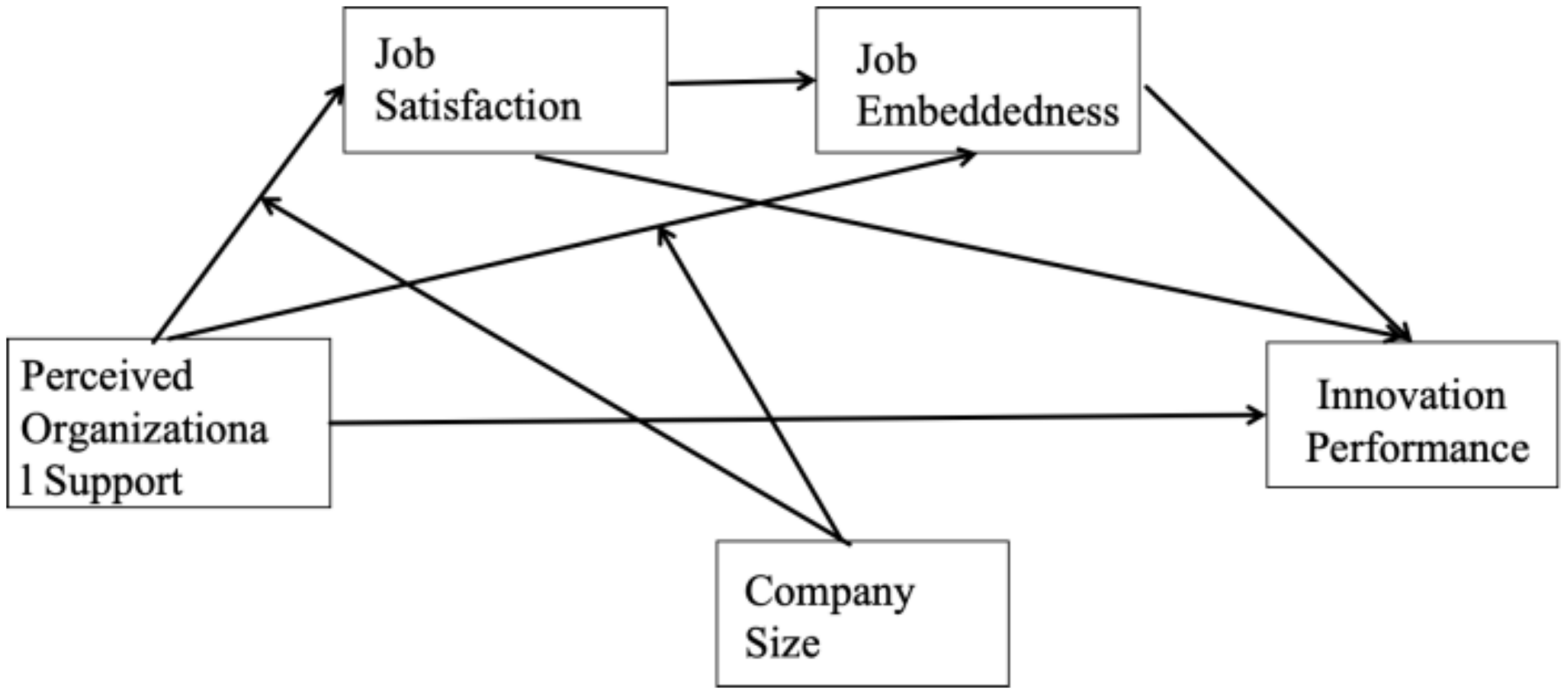
Research model. Source: Prepared by this study.

In summary, this research develops a model (refer to Figure 1) that examines how organizational support affects innovation performance of employees in AI companies.

## 3 Methods

### 3.1 Research process

**Figure 2:**
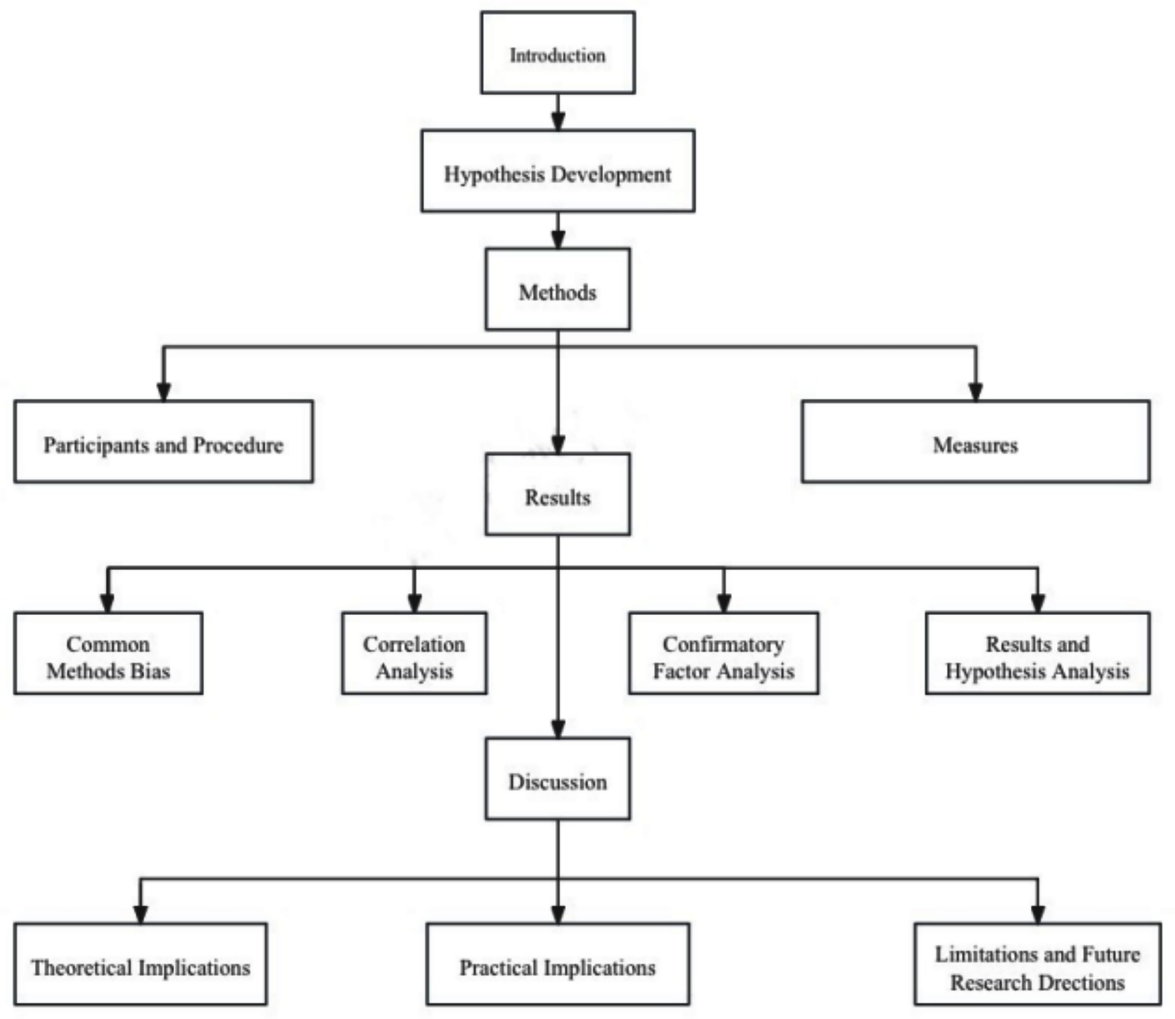
Process of this study. Source: Prepared by this study.

The overall research process of this study is summarized in Figure 2. Based on theoretical foundations such as Social Exchange Theory and Self-Determination Theory, the study develops hypotheses, determines the research methodology, conducts data analysis, and finally discusses theoretical and practical implications as well as research limitations.

### 3.2 Pre-test

To ensure measurement accuracy, this study conducted a pilot test among employees from two AI technology companies in Wuhan, ultimately collecting 89 valid responses. First, reliability and validity analyses were performed on the pilot test questionnaire. The results of the independent sample t-test indicated that all questionnaire items exhibited significant discrimination power. Additionally, the Cronbach’s *α* coefficients for the key constructs in this study—perceived organizational support (0.874), job satisfaction (0.897), job embeddedness (0.901), and innovation performance (0.890)—demonstrated high reliability. Second, the Kaiser-Meyer-Olkin (KMO) test was conducted to examine the construct validity of the questionnaire (Kaiser, 1974). The results confirmed that the validity fell within an acceptable range, indicating a good structural validity of the questionnaire. In conclusion, based on the pilot test results, the questionnaire meets reliability and validity requirements and is deemed suitable for the subsequent formal study.

### 3.3 Participants and procedure

This study collected data through an online survey platform, Wenjuanxing, which has extensive experience in questionnaire design and distribution. The target participants were employees from AI technology companies, and the survey was conducted on a voluntary and anonymous basis. Since this study examines whether the size of AI technology companies moderates the proposed model, AI firms were categorized into large, medium, and small enterprises based on the World Bank (2024) classification. Large enterprises are defined as companies with more than 300 employees and an annual revenue exceeding $100 million. Medium enterprises have between 50 and 300 employees with annual revenue ranging from $10 million to $100 million, while small enterprises have fewer than 50 employees and generate annual revenue below $10 million. To ensure a representative sample, the study randomly selected AI companies that met these criteria for data collection.

All participants were employees working in AI enterprises located in mainland China. The sample therefore represents a single national context, which ensures cultural and institutional consistency but may limit the generalizability of the findings to other countries or organizational environments.

The data collection process commenced on May 14, 2024, and lasted for 3 weeks, yielding a total of 640 questionnaire responses. To ensure data reliability and validity, the research team applied several screening criteria. First, to ensure data quality, the completion time was examined. The average time to complete the questionnaire was 12.3 min (SD = 3.1). Responses completed in less than 9 min—approximately one standard deviation below the mean—were excluded, as such short completion times are generally considered insufficient for careful reading and accurate responses (Meade and Craig, 2012). Second, response patterns were examined, and questionnaires in which all answers were identical were excluded. Third, logical consistency was checked, and any responses that contradicted themselves in reverse-coded items were also eliminated. After applying these criteria, 536 valid questionnaires were retained, resulting in an effective response rate of 83.75%.

The demographic analysis of the final dataset revealed that the sample consisted of 56.7% male and 43.3% female respondents. In terms of educational background, 41.8% of participants held a bachelor’s degree, while 58.2% had obtained a postgraduate degree or higher. Table 1 shows the demographic characteristics of the respondents.

**Table 1:** Demographics of respondents.

Demographics	Frequency	Percentage (%)
Gender		
Male	304	56.7
Female	232	43.3
Age		
Below 25 years old	106	19.78
25–32 years old	248	46.27
Above 32 years old	184	34.33
Company Size		
Large Enterprises	224	41.79
Medium Enterprises	202	37.69
Small Enterprises	110	20.52
Company type		
Algorithm/model development	140	26.1
Application-oriented	204	38.1
Platform/service providers	113	21.1
AI hardware firms	79	14.7

### 3.4 Measures

This study employed a five-point Likert scale to measure key variables:

(1) Perceived Organizational Support (POS): Employees’ perceived organizational support was measured using the 8-item short version of the *Perceived Organizational Support Scale* developed by Eisenberger et al. (1986). Items assess the extent to which employees believe their organization values their contributions and cares about their well-being. Responses were rated on a five-point Likert scale (1 = strongly disagree, 5 = strongly agree).

Example item: “My organization really cares about my well-being.”

The Cronbach’s *α* for this scale in the present study was 0.945, indicating excellent reliability.

(2) Innovation *Performance*: Individual innovation performance was measured using the *Individual Innovation Behavior Scale* developed by Scott and Bruce (1994). Although originally designed to assess innovation behavior, this scale has been widely adopted in subsequent studies as an indicator of individual innovation performance, as it captures the extent to which employees engage in idea generation, promotion, and implementation—core behavioral components that directly contribute to innovation outcomes (Janssen, 2000; Yuan and Woodman, 2010). In this study, the term “innovation performance” is used to emphasize the result-oriented aspect of these innovation-related behaviors within the AI enterprise context, where the successful transformation of ideas into applicable technologies and products constitutes a key measure of innovation effectiveness. The scale consists of six items rated on a five-point Likert scale (1 = strongly disagree, 5 = strongly agree).

Example item: “Searches out new technologies, processes, techniques, and/or product ideas.”

The Cronbach’s *α* coefficient for this scale ranged from 0.79 to 0.87, indicating satisfactory reliability.

(3) Job *Satisfaction*: Job satisfaction was measured using the *Job Satisfaction Scale (JSS)* developed by Anderson et al. (1951). The scale consists of five items rated on a five-point Likert scale (1 = strongly disagree, 5 = strongly agree), assessing employees’ overall satisfaction with their job.

Example item: “I feel fairly well satisfied with my present job.”

The Cronbach’s *α* coefficient for this scale was 0.87, confirming its reliability.

(4) Job *Embeddedness*: Job embeddedness was measured using the *Job Embeddedness Scale* developed by Crossley et al. (2007). The scale consists of seven items assessing employees’ perceived fit, links, and sacrifice within their organization. Responses were rated on a five-point Likert scale (1 = strongly disagree, 5 = strongly agree).

Example item: “I feel attached to this organization.”

This measure has been rigorously validated across diverse organizational contexts and demonstrates high reliability and construct validity. In the present study, the Cronbach’s α coefficient was 0.88, indicating strong internal consistency.

(5) Control Variables: Prior research has indicated that gender and age significantly influence employee *innovation* performance (Yu and Lee, 2017; Yu and Chen, 2016). Therefore, gender and age were included as control variables in this study.

## 4 Results

### 4.1 Common method bias

To assess potential common method bias, this study employed *Harman’s single-factor test*, a widely used diagnostic procedure for evaluating the presence of common method variance (Podsakoff et al., 2003). All items from the study’s measurement scales were entered into an unrotated exploratory factor analysis. The results indicated that the variance explained by the largest single factor was 24.02%, which is well below the commonly accepted threshold of 50%. This suggests that common method bias is not a significant concern in this study.

### 4.2 Correlation analysis

A correlation analysis was conducted to further explore the relationships between variables. According to the results presented in Table 2, several key findings emerged.

**Table 2:** Correlation analysis.

Variables	1	2	3	4	5	6	7
1. Gender	1						
2. Age	0.091	1					
3. Company Size	−0.124*	−0.043	1				
4. Perceived Organizational Support	0.179**	0.191**	−0.083	1			
5. Job Satisfaction	0.174**	0.193**	−0.131*	0.591**	1		
6. Job Embeddedness	0.206*	0.192**	−0.008	0.823**	0.643**	1	
7. Innovation Performance	0.214**	0.178**	−0.163**	0.423**	0.494**	0.477**	1

*p < 0.05, **p < 0.01. Source: Prepared by this study.

First, in AI technology enterprises, perceived organizational support (POS) was found to be positively correlated with job satisfaction (*r* = 0.591, *p* < 0.01) and job embeddedness (*r* = 0.823, *p* < 0.01). Additionally, POS was positively correlated with innovation performance (*r* = 0.423, *p* < 0.01). These findings provide preliminary evidence supporting the impact of POS and lay the groundwork for further exploring the mechanism between POS and innovation performance.

Second, from the perspective of innovation performance, both job satisfaction (*r* = 0.494, *p* < 0.01) and job embeddedness (*r* = 0.477, *p* < 0.01) exhibited positive correlations with innovation performance, suggesting that employees with higher job satisfaction and stronger job embeddedness tend to demonstrate greater innovation performance.

Finally, the analysis revealed that demographic variables such as gender and age had significant effects on the studied variables, indicating the necessity of controlling for these factors in further analyses.

### 4.3 Confirmatory factor analysis

This study employed Confirmatory Factor Analysis (CFA) using Mplus 7.4 to assess the discriminant validity of four key constructs: *perceived organizational support*, *job satisfaction*, *job embeddedness*, and *innovation performance*. The results demonstrated that the four-factor measurement model provided a good fit to the data (χ^2^/df = 2.54, RMSEA = 0.050, GFI = 0.901, CFI = 0.950, TLI = 0.961, IFI = 0.978).

The fit indices were evaluated according to the commonly accepted cut-off criteria proposed in the methodological literature. Specifically, a χ^2^/df value below 3.0, a Root Mean Square Error of Approximation (RMSEA) below 0.06, and Comparative Fit Index (CFI) and Tucker–Lewis Index (TLI) values above 0.90 are generally indicative of an acceptable model fit (Hu and Bentler, 1999; Kline, 2016). Similarly, a Goodness-of-Fit Index (GFI) and Incremental Fit Index (IFI) greater than 0.90 further support adequate fit.

Taken together, these results indicate that the measurement model achieves a satisfactory level of fit and provides strong evidence for the construct validity and discriminant validity of the variables used in this study.

### 4.4 Results and hypothesis analysis

This study first conducted a mediation model test using Model 6 in the SPSS macro developed by Hayes (Hayes, 2012). Controlling for gender and age, the study examined the mediating effects of job satisfaction and job embeddedness in the relationship between perceived organizational support and innovation performance. As shown in Table 3, perceived organizational support had a significant direct predictive effect on innovation performance (*b* = 0.235, *t* = 6.721, *p* < 0.001). However, after introducing the mediating variables, the direct predictive effect of perceived organizational support on innovation performance was no longer significant (*b* = 0.017, *t* = 0.304, *p* > 0.1), indicating that H1 was not supported.

**Table 3:** Regression results of the chain mediating effects model (*n* = 536).

Outcome variable	Predictive variable	R^2^	F	b	SEs	t	LLCI	ULCI
Equation 1								
Job Satisfaction	Perceived Organizational Support	0.36	49.516	0.394***	0.035	11.091	0.324	0.463
Equation 2								
Job Embeddedness	Perceived Organizational Support	0.718	167.12	0.434***	0.026	16.448	0.382	0.486
	Job Satisfaction			0.217***	0.038	5.728	0.142	0.291
Equation 3								
Innovation Performance	Perceived Organizational Support	0.302	22.684	0.017	0.057	0.304	−0.095	0.129
	Job Satisfaction			0.269***	0.061	4.431	0.149	0.388
	Job Embeddedness			0.216**	0.093	2.319	0.033	0.399

All estimated coefficients are unstandardized. Control variables of the regression model include gender and age. LLCI, Lower limit of the 95% CI, and ULCI, Upper limit of the 95% CI; and Number of bootstrap samples for percentile bootstrap confidence intervals is 5,000. **p < 0.05 and ***p < 0.01.

Further analysis of the mediating variables revealed that perceived organizational support had a significant positive predictive effect on job satisfaction (*b* = 0.394, *t* = 11.091, *p* < 0.001) and job embeddedness (*b* = 0.434, *t* = 16.448, *p* < 0.001), supporting H2 and H3. Additionally, job satisfaction had a significant positive predictive effect on job embeddedness (*b* = 0.217, *t* = 5.728, *p* < 0.001), confirming H6.

Regarding innovation performance, the results showed that both job satisfaction and job embeddedness had significant positive predictive effects on innovation performance (*b* = 0.269, *t* = 4.431, *p* < 0.001; *b* = 0.216, *t* = 2.319, *p* < 0.05), supporting H4 and H5. These findings suggest that the effect of perceived organizational support on innovation performance is realized through the mediating roles of job satisfaction and job embeddedness rather than through a direct effect.

Control variables (gender and age) were included in all regression models. The results indicated that neither gender nor age had a statistically significant effect on job satisfaction, job embeddedness, or innovation performance (*p* > 0.05), suggesting that these demographic factors did not materially affect the tested relationships. Therefore, their coefficients are not reported in Table 3 for simplicity.

Table 4 indicates that the total effect of the independent variable (perceived organizational support) on the dependent variable (innovation performance) is significant, with a total effect of 0.235. The Bootstrap confidence interval (BootLLCI = 0.166, BootULCI = 0.304) does not include zero, confirming the significance of the total effect. However, the direct effect is minimal, at only 0.017, and its confidence interval (BootLLCI = −0.095, BootULCI = 0.129) includes zero, suggesting that the direct impact of perceived organizational support on innovation performance is not significant.

**Table 4:** Results and comparison of chain mediating effect (*n* = 536).

	Effect	BootSE	BootLLCI	BootULCI	Ratio of indirect to total effect (%)
Total effect	0.235	0.035	0.166	0.304	-
Direct effect	0.017	0.057	−0.095	0.129	-
Total indirect effect	0.218	0.053	0.116	0.327	92.77%
Ind1: JSup→JSat→IP	0.106	0.023	0.059	0.152	45.11%
Ind2: JSup→JE→IP	0.094	0.043	0.014	0.185	40.00%
Ind3: JSup→JSat→JE→IP	0.018	0.010	0.002	0.041	7.66%
Ind1-Ind2	0.012	0.052	−0.095	0.112	-
Ind1-Ind3	0.087	0.026	0.035	0.138	-
Ind2-Ind3	0.075	0.035	0.011	0.15	-

Boot SE, Boot LLCI and Boot ULCI are estimated SE under bias-corrected percentile bootstrap method, and 95% CI lower and 95% CI upper, and Boot LLCI and Boot ULCI do not overlap with zero. JSup stands for Job Support. JSat stands for Job Satisfaction. JE stands for Job Embeddedness. IP stands for Innovation Performance.

Further analysis of the indirect effects reveals that the influence of perceived organizational support on innovation performance is primarily mediated through job satisfaction and job embeddedness. The total indirect effect is 0.218, with a Bootstrap confidence interval (BootLLCI = 0.116, BootULCI = 0.327) that does not include zero, indicating that the indirect effect is significant and accounts for 92.77% of the total effect. Moreover, job satisfaction plays the most dominant mediating role in the relationship between perceived organizational support and innovation performance, contributing 45.11% of the total effect.

Next, the moderation effect was tested using Model 7 in Hayes’ SPSS macro. Controlling for gender and age, company size was introduced as a moderator in the mediation model, forming a moderated mediation framework. The results are presented in Table 5.

**Table 5:** Testing of moderation effects (*n* = 536).

Variables	Job satisfaction				Job embeddedness			
	b	SEs	t	95%CI	b	SEs	t	95%CI
Perceived Organizational Support	0.385***	0.035	10.924	[0.316, 0.455]	0.523***	0.023	22.637	[0.477, 0.568]
Company Size	−0.098	0.058	−1.679	[−0.213, 0.017]	0.076**	0.04	1.998	[0.001, 0.152]
POS * CS	0.137**	0.062	2.230	[0.016, 0.259]	−0.011	0.041	−0.267	[−0.090, 0.069]
R^2^	0.377				0.687			
F	31.745				115.124			
ΔR^2^ (interaction)	0.034							

OS * CS stands for “Perceived Organizational Support* Company Size.” All estimated coefficients are unstandardized. Control variables of the regression model include gender and age. **p < 0.05 and ***p < 0.01.

The interaction term between perceived organizational support (POS) and company size significantly predicted job satisfaction (*b* = 0.137, SE = 0.062, *t* = 2.230, *p* < 0.05, 95% CI [0.016, 0.259]), whereas its effect on job embeddedness was not significant (*b* = −0.011, SE = 0.041, t = −0.267, *p* > 0.05, 95% CI [−0.090, 0.069]). The main effect of POS on job satisfaction remained significant (*b* = 0.385, SE = 0.035, 95% CI [0.316, 0.455]).

It should be noted that company size was coded inversely (1 = large, 3 = small). Therefore, the positive interaction coefficient (*b* = 0.137, *p* < 0.05) indicates that the positive relationship between perceived organizational support and job satisfaction is stronger in smaller companies and weaker in larger ones. Including the interaction term explained an additional 3.4% of the variance in job satisfaction (ΔR^2^ = 0.034, *p* < 0.05), supporting H7 but not H8.

**Figure 3:**
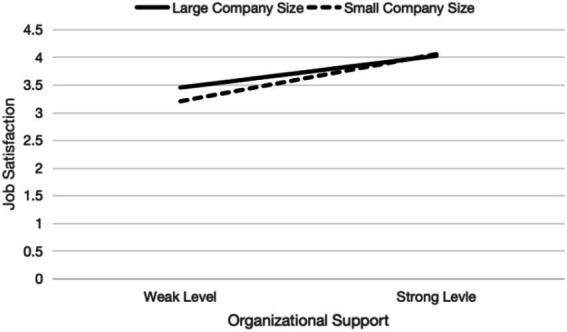
The moderating effect of company size. Source: Prepared by this study.

**Figure 4:**
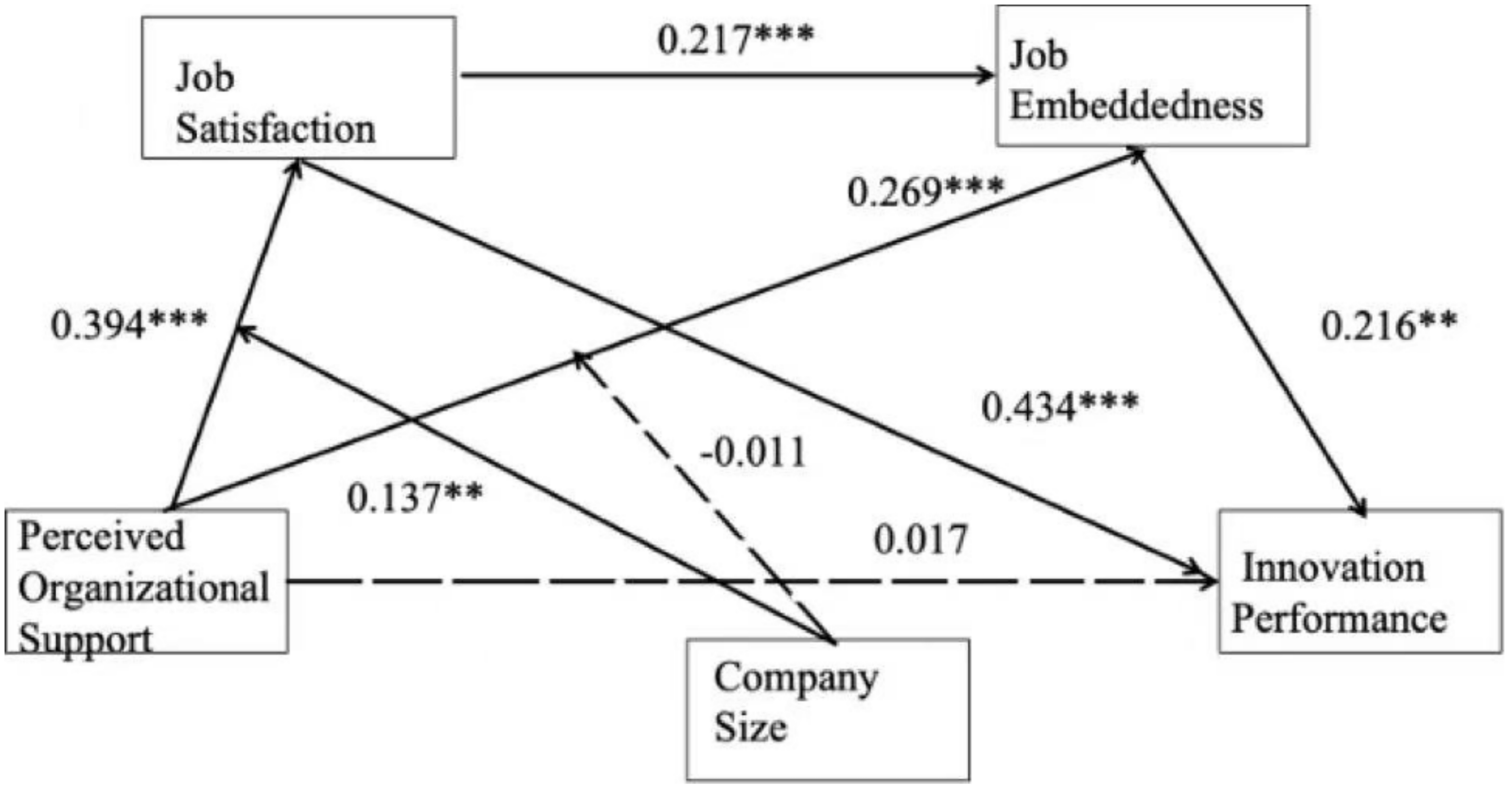
Path coefficients of the model. Source: Prepared by this study. **p < 0.05 and ***p < 0.01.

Simple slope analysis (Figure 3) further demonstrated that the effect of POS on job satisfaction became steeper for smaller firms (M + 1SD) and flatter for larger firms (M − 1SD). This suggests that employees in smaller organizations—characterized by flatter structures and closer interpersonal relationships—experience greater satisfaction gains from organizational support. The final research model is illustrated in Figure 4.

## 5 Discussion

### 5.1 Theoretical implications

First, we examined the impact of perceived organizational support (POS) on innovation performance within AI companies. Although numerous studies have demonstrated that POS significantly influences organizational innovation under the framework of Social Exchange Theory (SET; Richardson et al., 2008; Al-Taie and Khattak, 2024), most prior research has focused on traditional industries characterized by hierarchical structures, standardized procedures, and stable technological trajectories. In contrast, AI companies operate in volatile, knowledge-intensive environments, where innovation depends on rapid learning, algorithmic iteration, and interdisciplinary collaboration (Li et al., 2025). Unlike traditional firms—where innovation is often incremental and process-driven—AI enterprises must continually adapt to evolving data ecosystems and emergent technologies, requiring employees to engage in continuous cognitive renewal and proactive creativity. These conditions fundamentally reshape how organizational support is perceived and enacted. Consequently, this study addresses a significant gap by investigating how POS functions in the AI sector, revealing that—unlike in conventional industries—POS does not exert a direct effect on innovation performance. This aligns with emerging evidence that organizational support’s influence on innovation is indirect, operating primarily through motivational and attitudinal pathways rather than direct performance enhancement (Chu et al., 2024).

Second, we examined the mediating mechanisms linking perceived organizational support to innovation performance. Grounded in Social Exchange Theory and Self-Determination Theory (SDT), we propose that POS activates a reciprocal motivational process, whereby employees internalize organizational care as a signal of trust and value, leading to enhanced emotional attachment and identification with the organization. This, in turn, strengthens job satisfaction, reflecting the fulfillment of employees’ basic psychological needs for competence, autonomy, and relatedness (Ryan and Deci, 2020; McAnally and Hagger, 2024). Simultaneously, such satisfaction promotes job embeddedness, which captures the extent to which employees perceive fit with the organization, form social links, and recognize the sacrifices associated with leaving (Yamin, 2022; Yoon et al., 2022). Together, job satisfaction and embeddedness constitute a psychological nexus that translates perceived support into sustained commitment, discretionary effort, and innovative behavior. In this process, employees are not merely motivated by external rewards but by the internalized sense of belonging and purpose derived from supportive exchanges. Therefore, the findings of this study enrich existing knowledge by clarifying how these mediating variables jointly transmit the effects of POS to innovation performance, advancing both the theoretical and practical understanding of innovation management within AI contexts.

Finally, this study examined the moderating role of company size in the relationship between perceived organizational support (POS) and job satisfaction within the framework of Social Exchange Theory. The results indicate that organizational support enhances job satisfaction in both small and large AI companies, but the effect is notably stronger in smaller firms. This finding suggests that in smaller organizations, supportive actions are perceived more directly and personally, strengthening employees’ psychological reciprocity and emotional attachment, which in turn increases job satisfaction. Conversely, in larger firms, although structured systems and abundant resources exist, hierarchical distance and procedural rigidity may weaken the immediacy and emotional salience of organizational support.

These results align with prior empirical observations and offer theoretical clarification. Tansel and Gazioglu (2022) argued that the bureaucratic structures and limited flexibility of large firms can reduce employees’ autonomy and satisfaction, while smaller organizations tend to foster stronger social cohesion. Similarly, Inoue et al. (2024) demonstrated that perceived organizational support is generally lower in medium and large enterprises, but that supervisor support can mitigate this gap—underscoring that relational proximity amplifies the effectiveness of support mechanisms.

Together, these theoretical perspectives help explain why the effect of organizational support on job satisfaction is stronger in smaller AI firms.

From a theoretical standpoint, this finding refines the contextual boundary of Social Exchange Theory by showing that firm size moderates the strength of the POS–attitude linkage. Practically, it suggests that small AI companies should leverage close interpersonal exchanges to maintain high satisfaction levels, while large AI enterprises should improve the visibility, accessibility, and responsiveness of their support systems to sustain employee engagement and well-being.

### 5.2 Practical implications

In the context of intensified global competition and rapidly evolving technological environments, innovation has become a central determinant of organizational competitiveness and sustainable growth. The present study advances practical insights by demonstrating that perceived organizational support (POS) does not directly influence employees’ innovation performance, but rather exerts its effect through the mediating mechanisms of job satisfaction and job embeddedness. Furthermore, the moderating role of company size underscores the necessity for context-sensitive human resource management practices.

For small- and medium-sized enterprises, where organizational hierarchies are flatter and interpersonal connections are closer, managers should emphasize the creation of a psychologically supportive climate. Initiatives such as regular developmental feedback, open communication channels, and recognition programs that highlight employees’ contributions can strengthen perceptions of organizational care and reinforce job satisfaction. These relational practices, grounded in mutual trust and respect, are particularly effective in smaller organizational settings where informal interactions have a stronger impact on employees’ emotional attachment and engagement.

By contrast, large enterprises often face challenges stemming from structural complexity and hierarchical distance, which can weaken employees’ perception of support. For such organizations, formalized support systems are essential. Structured training programs, transparent promotion pathways, and cross-departmental innovation initiatives can communicate organizational commitment and enhance employees’ sense of embeddedness. These institutionalized mechanisms can compensate for the psychological distance created by organizational scale and contribute to sustained innovation performance.

Moreover, in the specific context of AI enterprises, management practices should leverage digital technologies to enhance employees’ perceived organizational support. Intelligent performance management platforms, AI-assisted mentoring systems, and data-driven feedback tools can provide timely recognition, personalized guidance, and transparent communication, thereby strengthening employees’ job satisfaction and embeddedness. Additionally, given the prevalence of remote or hybrid work structures in AI companies, regular virtual check-ins, open knowledge-sharing forums, and inclusive team rituals are essential to maintain employees’ sense of belonging and sustained engagement in innovation activities.

Overall, the findings emphasize that organizational support enhances job satisfaction in both small and large firms, but its influence is more pronounced in smaller organizations. This suggests that in smaller firms, supportive practices are more directly perceived and internalized by employees, resulting in stronger motivational and attitudinal outcomes. Hence, organizational support should be tailored to firm size, ensuring that managerial strategies are aligned with contextual characteristics to optimize employees’ innovative potential and sustain long-term organizational vitality.

### 5.3 Limitations and future research directions

Although this study provides meaningful insights into the mechanisms linking perceived organizational support, job satisfaction, job embeddedness, and innovation performance, several limitations should be noted.

First, the cross-sectional design restricts the ability to infer causality among variables; future studies could adopt longitudinal designs to strengthen causal explanations.

Second, the data were collected through self-report measures, which may involve certain subjective biases, though diagnostic tests indicated no serious common method bias.

Third, as the sample focused on AI enterprises in China, the generalizability of the findings to other industries and cultural contexts may be limited.

Finally, while the classic measurement scales used in this study have been extensively validated, future research could incorporate more updated and context-specific instruments to better capture employees’ perceptions in modern organizational environments.

## 6 Conclusion

To effectively enhance individual innovation performance, enterprises need to establish a supportive organizational environment that fosters job satisfaction and job embeddedness, thereby stimulating employees’ innovative potential. This study explores the impact mechanism of perceived organizational support on individual innovation performance, revealing the mediating roles of job satisfaction and job embeddedness, as well as the moderating effect of company size. The findings contribute not only to the theoretical advancement of organizational behavior and human resource management but also provide practical insights for AI enterprises in talent management and innovation incentives. By optimizing organizational support systems, companies can strengthen employees’ innovation motivation, drive continuous improvements in overall innovation capability, and maintain a competitive edge in an increasingly dynamic market environment.

## Data Availability

The raw data supporting the conclusions of this article will be made available by the authors, without undue reservation.
